# Emergency coronary artery bypass grafting using minimized versus standard extracorporeal circulation – a propensity score analysis

**DOI:** 10.1186/1749-8090-8-59

**Published:** 2013-04-02

**Authors:** Michael Ried, Assad Haneya, Philipp Kolat, Alois Philipp, Reinhard Kobuch, Michael Hilker, Christof Schmid, Claudius Diez

**Affiliations:** 1 Department of Cardiothoracic Surgery, University Medical Center Regensburg, Franz-Josef-Strauss-Allee 11, Regensburg, 93053, Germany

**Keywords:** Coronary artery bypass surgery, Minimized extracorporeal circulation, Emergency revascularization, Mortality, Propensity score analysis

## Abstract

**Background:**

The impact of minimized extracorporeal circulation (MECC) for emergency revascularization remains controversial.

**Methods:**

A total of 348 patients underwent emergency CABG with MECC (n=146) or conventional extracorporeal circulation (CECC; n=175) between January 2005 and December 2010. Using propensity score matching after binary logistic regression, 100 patients, who underwent CABG with MECC could be matched with 100 patients, who underwent CABG with CECC. Primary outcome was 30-day mortality.

**Results:**

Unadjusted 30-day mortality was 14.8% in patients with CECC and 6.9% in those with MECC (mean difference −7.9%; p=0.03). The adjusted mean difference (average treatment effect of the treated, ATT) after matching was −1.0% (95% CI −8.6 to 7.6; p=1.0). Intensive care unit stay (adjusted mean difference 1.0; 95% CI −0.2 to 3.2; p=0.70) and hospital stay (adjusted mean difference 1.0; 95% CI −2.0 to 3.6; p=0.40) did not show significant differences between both groups. The adjusted mean difference for postoperative low cardiac output syndrome was −1.1% (95% CI −7.3 to 7.1; p=0.83) without significant differences between CECC and MECC. Postoperative mechanical ventilation time, drain loss, postoperative rethoracotomy, postoperative neurological events, new onset renal replacement therapy and respiratory failure also had insignificant average treatment effects of the treated. In addition, all average treatment effects (ATEs) did not significantly differ between both groups.

**Conclusion:**

Using propensity score estimation and matching, we did not observe significant differences in terms of survival and further outcomes in patients who undergo emergency CABG with CECC or MECC, but our results call for further analysis.

## Background

Coronary artery bypass grafting (CABG) with cardiopulmonary bypass support (CPB) as standard perfusion technique remains the treatment of choice for high-grade left main-stem stenosis and triple-vessel coronary artery disease [1,2]. Several studies compared the impact of minimized versus conventional extracorporeal circuits and found conflicting results. Development and utilization of MECC have been proposed to reduce the adverse effects of CECC during CABG while taking the advantages of standard ECC [3-5]. Various publications have reported that MECC decreases perioperative side effects such as systemic inflammation, coagulation derangement and postoperative complications [6-8], whereas other reports failed to confirm these promising results [9]. Our department has been performing CABG with MECC for more than one decade and we could demonstrate that MECC is a safe alternative for on-pump and off-pump coronary revascularization [10]. Even in high-risk patients MECC using could be successfully applied [11].

However, emergency patients suffering from an acute coronary syndrome are at high-risk and perioperative mortality is still increased if undergoing CABG with standard ECC [12]. Recent studies reported a survival benefit in this high-risk group of patients by performing beating-heart or off-pump coronary revascularization [13-15]. Nevertheless, application of MECC in patients emergency CABG remains controversial and no evidence suggests using MECC for emergency CABG.

Purpose of the present study was to examine the feasibility and efficacy of minimized ECC use in patients undergoing emergency surgical revascularization. Propensity score matching reduced significant selection bias between both groups and enabled direct comparison.

## Methods

### Patients and study design

From January 2005 to December 2010 a total of 321 consecutive patients who underwent emergency coronary artery bypass graft surgery (CABG) at the University Medical Center Regensburg were studied. The study was approved by the university’s ethics board, the individual patients consent was waived because of the study’s retrospective design and data collection from routine care. Included were only patients with emergency presentation due to acute coronary syndrome (unstable angina, non-ST-segment and ST-segment elevation myocardial infarction, elevated myocardial markers) which prompted isolated CABG within 24 hours after hospital admission. Acute myocardial infarction was diagnosed by conventional 12-channel electrocardiography in addition to myocardial enzymes and confirmed by immediate coronary angiography. All patients were evaluated for percutaneous coronary intervention (PCI). Coronary lesions (left main-stem stenosis, complicated and multiple lesions) were unsuitable for primary PCI. CABG procedures were performed with conventional (CECC) or minimized extracorporeal circulation (MECC) under general anesthesia. Patients with severe aortic regurgitation, a body mass index (BMI) ≥ 35 kg × m^-2^ and renal end-stage failure were relative contraindications for MECC support. Complete surgical revascularization was the aim in all patients and hybrid procedures were not primarily indicated. Exclusion criteria were as follows: elective CABG, conversion to surgical correction after failed PCI, beating-heart techniques, off-pump revascularization (OPCAB), redo surgery and preoperative renal replacement therapy. All operations were performed by six senior surgeons who are experienced in CABG with conventional as well as minimized extracorporeal circulation.

### Data collection

Patients’ data were collected prospectively in and retrospectively extracted from the institution’s database and from medical records, including demographic, clinical and outcome data. Variables were defined according to the European System of Cardiac Operative Risk Evaluation (EuroSCORE) and perioperative mortality was predicted with the logistic version of this risk stratification model [16,17]. Serum creatinine (SCr) was measured preoperatively in mg/dL at the day of hospital admission. Postoperative the peak SCr value 24 hours after surgery was analysed. Glomerular filtration rate (GFR) was calculated with the abbreviated Modification of Diet in Renal Disease (MDRD) formula and expressed in mL/min/1.73 m^2^: MDRD-eGFR = 186.3 × SCr ^–1.154^ × age ^–0.203^[18]. Oliguria was defined as urine output < 500 mL within 24 hours. Postoperative acute kidney injury was defined as a decrease of glomerular filtration rate ≥ 50% or the need of dialysis due to oliguria. The diagnosis of pneumonia was based on clinical presentation, chest X-ray and positive sputum cultures. Postoperative respiratory failure was defined as severe decline of arterial pO_2_ < 50 mmHg, an increase in pCO_2_ > 60 mmHg and an increase in respiratory rate > 40/min. Postoperative central neurologic event included transient/prolonged ischemic neurological deficit and stroke.

### Study endpoints

The primary study endpoint was postoperative mortality defined as death within 30 days after operation. The secondary endpoint was postoperative morbidity during hospitalization, which were as follows: low cardiac output syndrome (LCOS), respiratory failure, prolonged mechanical ventilation, central neurologic event or requirement of renal replacement therapy.

### Surgical management and perfusion techniques

Full midline sternotomy was routinely done and all patients were operated on cardiopulmonary bypass by standard cannulation of the ascending aorta and the right atrium (two-stage cannula). Mild hypothermia was applied and complete cardiac arrest was induced using Calafiore’s blood cardioplegia. During conventional cardiopulmonary bypass (CPB) blood was collected in an open cardiotomy reservoir and afterwards transfused back to the patient. At the end of harvesting the left internal mammary artery (LIMA), heparin (350 IE/Kg) was administered to target an activated clotting time (ACT) of ≥ 450 seconds in patients with CECC. The MECC-system (Maquet, Rastatt, Germany) is a fully closed circuit without blood-air contact. The tube set was pre-connected and completely coated with heparin. The prime volume was 500 mL and no heparin was added. For MECC heparin (125 IE/kg) was administered after LIMA preparation targeting an ACT between 250 and 300 seconds because of the reduced artificial surface. Blood was saved only with cell saver. Distal anastomoses were done using 7–0 or 8–0 monofilament sutures. At the end of the operation heparin was antagonized with protamine sulfate.

Anesthesia was maintained with inhalational and intravenous agents. Postoperatively, all patients were transferred to the intensive care unit (ICU) and received standard monitoring and mechanical ventilation.

### Statistical analysis

Statistical analysis was done with Stata 10.1 SE (Stata Corp., College Station, USA). Figures were created either with Stata or SigmaPlot 12.5 (Systat Software Inc., San Jose, USA). Stata’s module *psmatch2*[19] was used for propensity score matching and covariate imbalance testing. Continuous variables were first tested for normality with the Shapiro-Wilk test and visually with Quantile-Quantile plots. If normally distributed, they are presented as mean ± standard deviation (SD), otherwise as median with interquartile range (25^th^ and 75^th^ percentile). Student’s t test was used for comparison of two continuous, normally distributed variables, whereas Wilcoxon’s ranksum test was taken for non-normally distributed variables. Categorical data were shown as frequency distributions and were analyzed with Fisher’s exact test for 2 × 2 tables or with the Chi-Square test.

Because patients in this study were not randomly assigned to CABG with MECC, we matched them based on their propensity (conditional probability) to undergo CABG with MECC. The propensity score (PS) is a subject’s probability of receiving a specific treatment conditional on the observed covariates. The PS was calculated by binary logistic regression including all variables marked with an asterisk in Table 1. Nearest neighbor matching with a caliper ε=0.25×σ_P_ (σ_P_ denotes standard deviation of the estimated PS) was used to match 100 patients in the MECC group to 100 patients from the CECC group (matching efficacy 68.5%; 100/146 of MECC patients; 100 pairs). We used non-replacement that is, one a treated case is matched to one non-treated case, both cases were removed from the pool. We defined logit=log((1-PS)/PS) as propensity score and used it for matching. The logit of PS is called linear predictor of the PS.

**Table 1:** Unadjusted, i.e. before matching, pre- and operative data

**Variable**	**CECC (n=175)**	**MECC (n=146)**	**p-value**
Age [years]	68.4 ± 9.74	67.8 ± 8.74	0.55
Age group [n; %]			
<59	32	25	0.59
60 -69	64	54	
70-79	56	54	
>80	23	13	
Male gender [n; %]	129; 73.7%	113; 77.3%	0.44
Logistic EuroSCORE [%, 95% CI]	16.0 (13.4 to 18.2)	12.8 (10.9 to 14.6)	0.07
Ejection fraction [%]	50 (40; 64)	56 (45; 70)	0.001*
Height [cm]	172 (165; 176)	172 (166; 176)	0.65
Weight [kg]	82 (73; 93)	80 (70; 90)	0.11
Atrial fibrillation, preoperative [n; %]	9; 5.1%	6; 4.1%	0.66
COPD, preoperative [n; %]	17; 9.7%	9; 6.1%	0.24
Inhalative β_2_-Mimetic use [n; %]	5; 2.9%	4; 2.7%	0.94
Myocardial infarction preoperative [n; %]	140; 80%	116; 79.4%	0.90
Troponin I preoperative [ng/mL)^A^	5.1 (1.0 to 21)	3.8 (0.4 to 0.7)	0.15
Insulin-dependent diabetes [n; %]	15; 8.6%	11; 7.6%	0.73
Non insulin-dpendent diabetes [n; %]	26; 14.8%	21; 14.3%	1.0
Diabetic nephropathy [n;%]	9, 5.1%	5; 3.4%	0.45
Serum creatinine, preoperative [mg ×dL^-1^]	1.0 (0.8; 1.3)	0.9 (0.8; 1.2)	0.05*
Estimated GFR < 60 mL × min^-1^ × 1.73 m^-2^ [n; %]	63; 36%	32; 22%	0.006
No of grafts [n]	2.95 ± 0.88	2.7 ± 0.80	0.006*
LIMA use [n; %]	126; 72%	121; 83%	0.02*
Bypass time [min]	96 (69; 119)	74 (54; 93)	< 0.0001*
Aortic cross clamp time [min]	47 (34; 61)	37 (27; 50)	0.0002*

^A^ – Reference values (0.01 ng/ mL to 0.1 ng/mL).

Before matching the mean PS for MECC use in patients operated with CECC (n=175) was 0.399 ± 0.168 and in those receiving CABG with MECC 0.524 ± 0.156 with an associated standardized difference of 77.1% (95% CI 54 to 99; t test p-value < 0.0001). After matching, the mean PS for MECC use in the matched patients not receiving MECC was 0.471 ± 0.144 and in those receiving MECC was 0.474 ± 0.135 which yielded a standardized difference of 2.1% (95% CI −2.6 to 2.9; p > 0.05 for a two-tailed test).

**Figure 1:**
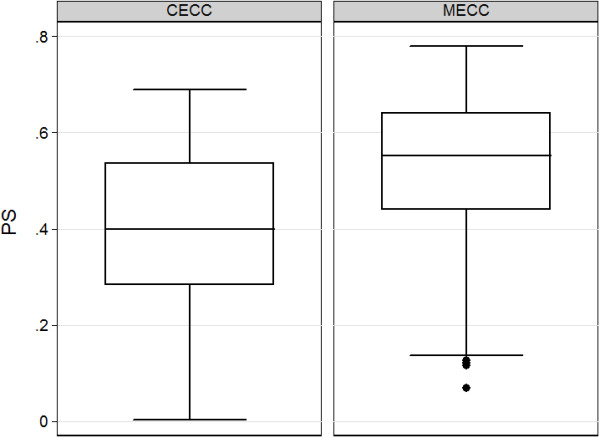
**Box plot of estimated native propensity score (not the logit of PS) stratified to type of extracorporeal circulation.** There is a sufficient overlap of propensity scores between both groups. MECC, Minimized extracorporeal circulation; PS, propensity score.

Figure 1 displays the distribution of estimated propensity scores stratified to treatment (MECC) or control (CECC).

Covariate imbalance was tested using the standardized difference before and after matching together with the achieved percentage reduction in absolute bias. The standardized difference is the difference of the sample means in the treated and non-treated sub-samples as a percentage of the square root of the average of the sample variances in the treated and non-treated groups.

The average treatment effect for the treated (ATT) answers the question: Is MECC beneficial for those individuals, who were (not randomly) assigned to treatment, i.e. MECC and was estimated with Wilcoxon’s signed rank test for continuous variables or with McNemar’s test for risk differences to account for the paired data structure after matching. We also calculated the average treatment effect that answers the question if MECC is beneficial for those randomly drawn from the overall population. Bootstrapping (400 replications) was used to estimate 95% confidence intervals (CIs) for ATEs. The key notion is ATT≠ATE and they are linked via ATE = N_1_/N × ATT + N_0_/N × ATU (N_0_ denotes the number of non-participants, N_1_ denotes the number of participants and ATU is the average treatment effect for the non-participants.

Hidden bias was estimated according to Rosenbaum’s sensitivity analysis. We used Stata’s *rbounds* module [20] for this test.

## Results

### Patient characteristics

Unadjusted baseline data before matching were summarized in Table 1. Several pre- and operative data were significantly different between both groups, including ejection fraction, renal function and LIMA use. It may reflect the non-randomized study design. Whereas EF was slightly higher in the MECC group, bypass and cross-clamp time were longer in the CECC group.

After matching, treated patients were similar in terms of all baseline covariates used for PS estimation (Table 2).

**Table 2:** Covariate balance testing between unmatched and matched sample

**Variable**	**Standardized difference [%]**		**% reduction |difference|**	**p-value**	
	**Unmatched**	**Matched**		**Unmatched**	**Matched**
Ejection fraction	44.6	0.3	99.4	0.02	0.98
Serum creatinine	−18.3	8.3	54.6	0.14	0.28
No of grafts	−26.5	22.1	16.6	0.03	0.11
LIMA use	31.1	2.6	91.5	0.01	0.85
Bypass time	−66.3	3.7	94.5	< 0.0001	0.78
Cross clamp time	−34.1	8.9	73.9	0.005	0.52

Matching reduced covariate imbalance and improved covariate balance between treatment groups. The median absolute bias before matching was 32.6% and after matching 5.9%.

### Outcome

The unadjusted 30-day mortality was 14.8% (26/175) in the CECC group and 6.9% (10/146) in the MECC group (mean difference −7.9%; p=0.032). After matching, the mean difference was −1.0% (95% CI −8.6 to 7.6; p=1.0) and shows no survival benefit for emergency patients, who were primarily assigned to MECC. If randomly drawn from the overall population, emergency CABG with MECC also does not exert a survival benefit (ATE; -1.5%; 95% CI −8.1 to 5.8; p > 0.05).

Table 3 summarizes the results for the secondary outcome variables ICU-stay, hospital stay and postoperative low cardiac output syndrome. Both ATT and ATE for all three variables failed to demonstrate benefits for MECC.

**Table 3:** Estimated average treatment effects on several outcome variables

**Comparison**	**Outcome measures**			
**ATT**^**A**^	**30-day mortality [%]**	**ICU-stay [days]**	**Hospital stay [days]**	**Low cardiac output [%]**
CECC versus MECC				
CECC (n=175)	14.8	5.3	13	11.4
MECC (n=146)	6.9	4.6	12	5.6
Unadjusted mean difference	−7.9	−0.8	−1	−5.8
p-value	0.03^B^	0.11	0.80	0.07^B^
Adjusted mean difference after matching of 100 pairs (95% bias corrected CI)	−1.0 (−8.6 to 7.6)	1.0 (−0.21 to 3.24)	1.0 (−2 to 3.6)	−1.1 (−7.3 to 7.1)
p-value	1.0^C^	0.70^F^	0.40^F^	0.83^C^
ATE^D^				
Adjusted mean difference with 95% bias corrected CI)	−1.5 (−8.1 to 5.8)^E^	0.61 (−0.7 to 2.2)^E^	0.34 (−2.5 to 2.6)^E^	−1.7 (−7.1 to 6.5)^E^

^A^ – ATT Average treatment effect for the Treated.

^B^ – Fisher’s exact test.

^C^ – McNemar’s test.

^D^ – ATE Average treatment effect.

^E^ – The 95% bias corrected confidence interval does include a zero, or p > 0.05 for a two-tailed test.

^F^ – Wilcoxon’s signed rank test.

Unadjusted in-hospital mortality was 13.7% (24/175) in the CECC group and 5.5% (8/146) in the MECC group (mean difference – 8.8%; p=0.015). The ATE was estimated with −1.1% (95% CI −7.1 to 6.4; p=1.00) and the ATE was estimated with −1.0% (95% CI −8.1 to 3.8).

Other outcome measures included postoperative ventilation time, drain loss, frequency of rethoracotomy and incidence of in-hospital neurologic event, new onset temporary renal replacement therapy (RRT) and incidence of respiratory failure. Before matching, the median postoperative ventilation time was 14 (9; 60) hours in the CECC group and 12 (8; 20) hours in the MECC group (p=0.05). After matching the average treatment effect of the treated (ATT) was 20 hours (95% CI −2.4 to 72; p > 0.05) showing no benefit for those, who were primarily assigned to MECC. The ATE was 13.2 hours (95% CI −16.8 to 48.7; p > 0.05). Table 4 displays the results of the average treatment effects for the treated and average treatment effects for the remaining outcome variables. Since all confidence intervals for both ATT and ATE include a zero (p > 0.05 for a two-tailed test), there is no significant difference between MECC and CECC after matching to be expected. In other words, emergency patients with identical risk and revascularization have similar outcomes after CABG irrespective CECC or MECC use.

**Table 4:** Secondary outcome measures

**Variable**	**Unadjusted**			
	**CECC**	**MECC**	**ATT (95% CI)**	**ATE (95% CI)**
Drain loss in 24 h [mL]	625 (350; 1100)	600 (350; 1000)	161 (−111 to 464)	164 (−109 to 406)
Rethoractomy [%]	9.7	8.9	3.2 (−4.9 to 12.1)	1.5 (−6.2 to 9.3)
Neurological event [%]	4.6	1.4	4.3 (−1.0 to 1.2)	−4.0 (−8.6 to 2.3)
New onset RRT [%]^A^	8.6	5.5	2.1 (−5.8 to 8.2)	2.5 (−3.2 to 8.5)
Respiratory failure [%]	15.4	13.0	3.2 (−2.4 to 14.9)	1.5 (−4.3 to 14.3)

^A^ – RRT Renal replacement therapy.

The frequency of rethoracotomy was almost 10% in both groups and the most common reason was postoperative bleeding (80%) followed by hematoma. Postoperative low cardiac output was treated either with intra-aortic ballon pump (n=2 in the MECC group; n=4 in the CECC group) or with veno-arterial ECMO (n=8 in the CECC group).

### Sensitivity analysis

Selection bias remains the most challenging analytic problem in observational studies and thus, we conducted a sensitivity analysis using Rosenbaum’s approach. Using Wilcoxon’s signed rank test, the sensitivity analysis showed that our study becomes sensitive to hidden bias at Γ = 1.8 for 30-day mortality, at Γ = 1.5, at Γ = 1.2 and at Γ = 2.5 for low cardiac output syndrome. Because these values are small, we conclude that the study is quite sensitive to hidden bias and therefore further analysis that control for additional biases is warranted.

## Discussion

Emergency CABG should be performed in patients with cardiogenic shock due to left main or severe triple vessel disease and whenever PCI is unlikely to achieve complete revascularization [21]. However, the application of different revascularization techniques to patients undergoing emergency CABG remains controversial. Data on optimal treatment strategies are still lacking and the logistic EuroSCORE as a tool for risk stratification is suggested to overestimate mortality in emergency patients [22]. Emergency procedures are associated with an enhanced perioperative risk, mainly caused by patient’s preoperative condition. Using ECC with cardioplegic arrest may contribute to surgical complications and mortality [12]. Some previous studies reported improved outcome for those patients operated upon beating heart or off-pump [14,15,23] with mortality rates comparable to patients operated upon on-pump [24].

To the best of our knowledge, there are no studies that examine the impact of minimized circuits on outcome after emergent CABG surgery compared to conventional ECC. Propensity score methods are increasingly being used to reduce the impact of treatment-selection bias in the estimation of causal treatment effects in observational studies [25,26]. We used propensity score matching to estimate the treatment effect of MECC for emergency CABG procedures because the comparison of the unadjusted data showed significant bias. After matching we failed to demonstrate benefits for MECC in terms of survival, ICU and hospital stay, LCOS, postoperative ventilation, new onset renal replacement therapy among several others. Although our primary sample comprised only 321 patients and we achieved a moderate matching efficiency of 69%, the resulting pairs were very comparable with a very low standardized difference of the PS.

The unadjusted data indicate an imbalance in patient selection, i.e. MECC patients had a better EF, a lower number of grafts, a somewhat higher LIMA use and shorter bypass and cross-clamp times. Thus, improved outcome may be expected. A question of concern arose from the overall low LIMA use and it was not possible to address this issue entirely. Analysis of patient records mentioned hemodynamic instability in majority of patients and inappropriateness of vessel diameter or flow in some cases. In the minority of cases, the decision to use LIMA or not, however, was an individual surgeon decision but gives room for improvement.

In non-emergency patients, MECC was shown to exert some benefit in terms of postoperative renal function, transfusion requirement and neurological dysfunction [27,28]. We failed to show these effects in our matched cohort since new onset RRT and incidence of cerebral event did not differ. We did not specifically address the issue of transfusion requirement because the majority of our patients received multiple anticoagulants before surgery and usually, RBC and plasma transfusions are more frequently seen in this patient group.

However, even advanced statistical methods to adjust imbalance between control and treatment in observational studies cannot compensate the current lack of at least one sufficiently powered randomized multicenter trial to estimate outcomes of emergency CABG with MECC and CECC. The mechanism why MECC might be associated with reduced mortality is still speculative and ranges from improved myocardial protection through consequent use of blood cardioplegia, reduced transfusion requirement, less pronounced inflammatory response, to selection of healthier patients. Critics of minimized ECC also may question whether small clinical effects justify a more complex procedure with learning curves and more intense and challenging interplay between surgeon, anesthesiologist and perfusionist.

The unadjusted 30-day mortality, particularly in the CECC group appears quite high. However, other studies also report data of approximately 10% [23] and 11.4% [29] and thus our reported total mortality of 11.8% is in line with these reports, but offers room for improvement. Since after matching the discrepancy between both groups almost disappeared, it seems that some patients in the CECC group had unmeasured comorbidity that might have contributed to excessive mortality.

The interplay between proper patient selection, pre- and perioperative stabilization including medical treatment and optimal surgical technique remain mandatory to address this high-risk patient group.

### Strength and limitations

Findings from propensity score analyses might be potentially limited by biases related to unmeasured and hidden covariates. Since our sensitivity analysis showed low values for Γ for several outcome variables, it is likely that unmeasured covariates contribute to our results and require further research. Incomplete or inexact matching might also affect study results. Our matching efficiency was almost 70% of all MECC patients, but still contrasts other PS studies with matching efficiencies < 50%. On the other hand, those matched were very comparable and enabled direct analysis.

## Conclusions

We could not demonstrate significant different treatment effects for patients undergoing emergency revascularization with minimized or conventional extracorporeal circulation. The findings of our study, based on a non-randomized design, are largely hypothesis generating and call for at least one randomized, multicenter trial using a current risk score for precise uniform evaluation of coronary pathology and predefined criteria for perioperative care. Thus, routine MECC use for emergency CABG currently may not be indicated.

## Competing interests

The authors declare that they have no competing interests.

## Authors’ contributions

MR made substantial contributions to the design of this study, acquisition of data, data analysis and interpretation of data. MR wrote and revised the manuscript. AH, PK, AP, RK, MH and CS participated in the design of this study and have been involved in revising the manuscript critically for important intellectual content. CD made substantial contributions to the design of this study and its coordination. CD helped with data analysis and interpretation of data as well as revising the manuscript critically for important intellectual content. CD has given final approval of the version to be published. All authors read and approved the final manuscript.
